# Stability of Operating Noise for Packaged MEMS Gyroscopes in Low-Speed Vehicle Motion Scenarios

**DOI:** 10.3390/mi17080947

**Published:** 2026-08-08

**Authors:** Xu Yang, Yanshun Zhang, Zhaoyang Liu, Yajuan Wang, A-Ni Li, Yang Pang

**Affiliations:** 1 School of Intelligent Mechatronics, Shaanxi Energy Institute, Xianyang 712000, China; yangxu@buaa.edu.cn (X.Y.);; 2 Faculty of Electronic and Information Engineering, Xi’an Jiaotong University, Xi’an 710049, China; 3 School of Instrumentation Science and Opto-Electronics Engineering, Beihang University, Beijing 100191, China; 4 School of New Energy, Shaanxi Energy Institute, Xianyang 712000, China

**Keywords:** MEMS, gyroscope, low-speed vehicle, angle random walk, Allan Variance, power spectral density, bias stability

## Abstract

Micro-Electro-Mechanical System (MEMS) gyroscopes serve as core components for attitude-sensing systems in low-speed unmanned vehicles and mobile robots. The long-term dynamic stability of MEMS gyroscopes under actual continuous low-speed vehicle operation is significantly inferior to the nominal performance derived from laboratory-based static calibration. This paper adopts a navigation-grade fiber optic gyroscope as the high-precision angular velocity reference. Angular velocity error sequences between the MEMS gyroscope and fiber optic gyroscope are established using field test data collected from a low-speed vehicle experiment lasting approximately 3.3 h. Three analytical approaches are applied to systematically characterize noise evolution features of the MEMS gyroscope under static and dynamic conditions from multiple dimensions. These approaches include time-domain drift analysis, angle random walk evaluation via Allan Variance, and frequency-domain interpretation based on Welch power spectral density. The test results reveal that vehicle motion significantly degrades the 10 s averaged bias stability of the MEMS gyroscope. The bias stability values under dynamic conditions increase by 2.1, 2.7 and 7.7 times compared with static states respectively. Root mean square analysis through band segmentation integration of Welch power spectral density indicates that vehicle motion induces the most obvious rise in noise energy in the middle frequency band. The amplification factor of noise along the *Y* axis reaches 24.7 times. The joint analytical framework proposed in this paper takes fiber optic gyroscope measurements as the reference and integrates time-domain analysis, Allan Variance and frequency-domain methods. It can provide sufficient experimental evidence and technical support for dynamic error compensation of MEMS gyroscopes deployed on low-speed mobile platforms.

## 1. Introduction

Low-speed mobile platforms such as automatic delivery vehicles and park inspection robots have been widely deployed in smart logistics, park security and public service sectors [1,2]. Such equipment exhibits typical operating characteristics. These features include low travel speed, long continuous working duration, frequent start–stop operations, and frequent low-speed steering maneuvers. The attitude sensing system acts as the fundamental core for navigation positioning and motion control of mobile platforms. The long-term operational stability of sensing devices directly determines the precision of carrier attitude maintenance, heading retention and trajectory tracking performance. It also serves as a critical factor restricting the overall operational reliability of low-speed intelligent mobile equipment [3,4].

Packaged MEMS gyroscopes have become the dominant attitude-sensing devices for low-speed mobile platforms in medium- and low-precision inertial attitude measurement fields [5,6,7]. They feature compact size, ultra-low power consumption and low cost. The inherent manufacturing structure and internal electromechanical coupling effects lead to complex error mechanisms for MEMS gyroscopes [8]. The total error consists of multiple components. These components include slow bias drift, angle random walk, rate random walk, vibration coupling error and environment-sensitive error. Each type of error follows distinct evolution rules under different operating conditions [9,10].

Current performance evaluation, parameter calibration and error modeling of MEMS gyroscopes in industrial communities are mostly implemented under static environments [11,12]. Device datasheet parameters and conventional calibration tests only obtain fixed noise coefficients under stationary conditions. These static parameters are directly adopted for navigation filter design and error compensation algorithm development. External disturbances, including road excitation, vehicle suspension vibration and steering maneuvers alter the high-frequency noise floor of sensors in practical continuous low-speed vehicle operation [13,14]. Low-frequency motion components generated by long-duration vehicle maneuvers and slow turns couple closely with the inherent bias drift of gyroscopes. This phenomenon makes the actual working errors of MEMS gyroscopes deviate greatly from static nominal specifications. Static calibration parameters alone fail to accurately predict the service performance of MEMS gyroscopes under vehicle dynamic conditions. This limitation further triggers engineering problems such as degraded integrated navigation accuracy and invalid control algorithms.

The analysis of device noise characteristics represents a classic research topic in the field of inertial navigation [15,16]. The Allan Variance method can separate multiple types of random noise and quantify noise intensity. It has evolved into a standard technique for gyro random error identification. This method is widely adopted in the performance test of various inertial sensors including fiber optic gyroscopes, laser gyroscopes and MEMS gyroscopes [17,18,19,20]. Numerous existing studies calculate key parameters of MEMS gyroscopes through the Allan Variance method under static test conditions. These parameters cover angle random walk, bias instability, and rate random walk. A mature workflow for static error modeling has therefore been established.

Several scholars have further explored the influences of single environmental variables, such as vibration and temperature, on MEMS gyroscope errors. Relevant studies have verified that external excitations lead to the degradation of gyroscope noise performance [21,22,23]. Current research still has obvious limitations. Most experiments rely on simulated environments including vibration tables and temperature control chambers. These setups cannot reproduce complex coupled dynamic conditions during the actual running of low-speed vehicles. Typical working conditions include vibration, steering and frequent start–stop actions. Some existing studies fail to effectively decouple the real angular motion of the carrier from inherent sensor errors. Direct analysis of original output data from MEMS gyroscopes may mistake vehicle attitude motion for sensor noise. This problem distorts the final error analysis results. No multidimensional joint evaluation framework combining time-domain, frequency-domain and random noise characteristics has been proposed for long-term continuous operation scenarios of low-speed vehicles. The evolutionary mechanism of dynamic noise in MEMS gyroscopes cannot be fully revealed with existing research schemes.

Fiber optic gyroscopes are high-precision inertial sensors characterized by extremely low static noise and excellent long-term bias stability [24,25]. These sensors can serve as the angular velocity reference for packaged MEMS gyroscopes. Carrier motion information can be separated from inherent errors of MEMS gyroscopes with this reference. This technical idea provides a feasible solution for accurate dynamic noise analysis of MEMS gyroscopes. Few relevant practical cases have been reported at present. Systematic experimental verification and mechanism analysis targeting low-speed mobile platforms remain insufficient.

To address the limitations of existing studies and satisfy practical engineering demands, this paper conducts an in-depth investigation into dynamic noise stability of MEMS gyroscopes under external excitations including vehicle-borne vibration and steering maneuvers generated during long-duration continuous motion of low-speed vehicles. The major works and contributions are summarized as follows:

(1) **Dynamic error decoupling for operational conditions:** A navigation-grade FOG serves as the field motion reference benchmark. Real-time decoupling is realized between true angular motion of the carrier and inherent errors of MEMS gyros, breaking the bottleneck that conventional experiments fail to reproduce in actual coupled operating conditions.
(2) **Time-varying dynamic Allan Variance analysis:** A sliding-window improved Allan Variance scheme targeting sustained dynamic operation is proposed, which reveals the time-varying evolution rules of noise parameters such as angle random walk against runtime and motion status.
(3) **Quantitative characterization of band-split noise energy:** The Welch power spectral density method combined with band-divided root mean square calculation is adopted to quantify differentiated amplification laws of noise energy across all frequency bands from static to dynamic states.
(4) **Comprehensive evaluation framework:** Integrating time-domain drift analysis, sliding-window DAVAR and band-segmented PSD analysis, a dynamic reliability evaluation system for MEMS gyros is constructed to fit practical operating conditions of low-speed mobile platforms.

## 2. Methodology and Models

### 2.1. Error Decoupling Model

The angular velocity output of the MEMS gyroscope under dynamic operating conditions contains both real carrier motion information and inherent sensor errors. The measured angular velocity of a three-axis gyroscope can be expressed as follows.

(1) $$\omega_{m}\left(t\right)=\omega_{v}\left(t\right)+b\left(t\right)+n\left(t\right)+d\left(t\right)$$

where $\omega_{v}(t)$ represents the actual angular velocity of the vehicle. b(t) stands for the slowly varying bias component of the gyroscope. n(t) denotes the random noise of the sensor. d(t) is the dynamic interference term induced by vibration structural coupling and environmental disturbances.

Conventional dynamic field experiments only acquire the integrated output signal of gyroscopes. They cannot effectively separate the above components. Direct Allan Variance analysis based on original gyroscope measurement data will produce random parameters disturbed by vehicle maneuvers. These parameters fail to truly reflect the inherent error characteristics of the sensor. An external angular velocity reference device with higher precision is required to independently analyze the intrinsic sensor errors.

A navigation-grade fiber optic gyroscope is used as an independent reference for carrier motion. The random noise of the fiber optic gyroscope is much lower than that of the MEMS gyroscope within the target analysis frequency band. The measurement output of the fiber optic gyroscope can therefore be approximated as follows.

(2) $$\omega_{f}\left(t\right)=\omega_{v}\left(t\right)+\varepsilon_{f}\left(t\right)$$

where $\varepsilon_{f}(t)$ refers to the residual error of the fiber optic gyroscope itself.

The error sequence of the MEMS gyroscope is constructed accordingly as:

(3) $$e\left(t\right)=\omega_{m}\left(t\right)-\omega_{f}\left(t\right)$$

The simultaneous expansion of these formulas yields the following result:

(4) $$e\left(t\right)=b\left(t\right)+n\left(t\right)+d\left(t\right)-\varepsilon_{f}\left(t\right)$$

The residual error of the fiber optic gyroscope satisfies the following condition:

(5) $$\left|\varepsilon_{f}\right|\ll \left|b+n+d\right|$$

**Figure 1:**
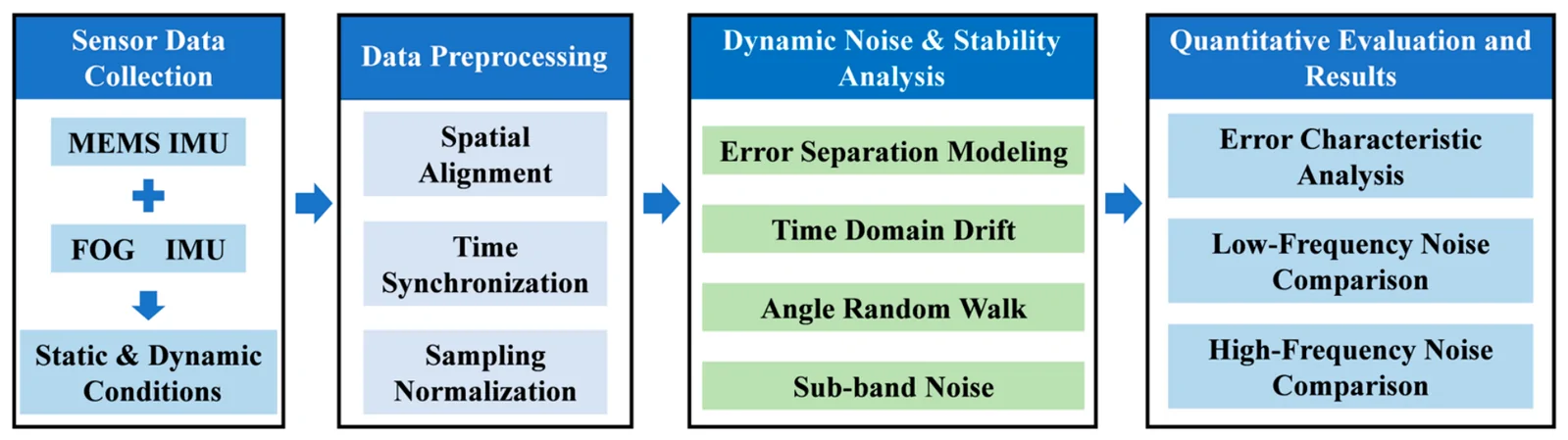
The proposed dynamic analysis and evaluation method.

This difference sequence can effectively characterize the inherent errors of the MEMS gyroscope. Compared with traditional methods that directly analyze raw outputs, the proposed scheme eliminates the real rotational component of the carrier. It supports accurate research on the evolution law of sensor random errors. The detailed methodological procedure is illustrated in Figure 1.

### 2.2. Time-Domain Drift and Bias Stability

Time-domain analysis mainly covers two indicators including long-term cumulative attitude drift and bias stability. These two indicators are adopted to characterize the slowly varying characteristics and statistical stability of MEMS gyroscope errors over time [26,27,28].

#### 2.2.1. Long-Term Cumulative Attitude Drift

The integral of angular velocity error over time yields cumulative attitude drift $\theta_{d}$. This index intuitively reflects the cumulative effect of errors over time t. The integral formula is given as follows.

(6) $$\theta_{d}\left(t\right)=\int_{0}^{t}e\left(t\right)dt$$

The cumulative drift curves under static and dynamic segments are extracted respectively. The least squares method is adopted for linear fitting to calculate drift slope and coefficient of determination. The drift slope reflects the long-term average drift trend of the gyroscope. The coefficient of determination evaluates the linear correlation degree of drift.

#### 2.2.2. Bias Stability in Static and Dynamic Conditions

Bias stability serves as a core indicator for evaluating the low-frequency error of gyroscopes. It is generally assessed via the N-second averaging method [29,30]. A 10 s averaging method is adopted in this paper. The angular velocity error sequence is smoothed using non-overlapping sliding average windows, followed by standard deviation calculation. The 10 s bias stability $\sigma_{b,i}$ of the three-axis MEMS gyroscope can be expressed as follows.

(7) $$\sigma_{b,i}=std\left({\bar{e}}_{i,k}^{10s}\right),i\in x,y,z$$

where ${\bar{e}}_{i}^{10s}$ denotes the average angular velocity error of the *i*-th axis within each 10 s time window. The unified duration of averaging windows avoids incomparable evaluation indicators caused by inconsistent filtering scales.

To quantitatively describe the performance degradation degree of the gyroscope caused by vehicle movement, the dynamic degradation ratio is defined as the ratio of bias stability under dynamic operating conditions to that under static operating conditions. A larger degradation ratio $D_{i}$ indicates that the corresponding axis is more severely affected by vehicle motion.

(8) $$D_{i}=\frac{\sigma_{b,i}^{dynamic}}{\sigma_{b,i}^{static}}$$

Meanwhile, to analyze the variation rule of bias stability with different averaging time scales, static and dynamic bias stability values under multiple window durations including 1 s, 2 s, 5 s, 10 s, 20 s, 50 s and 100 s are calculated sequentially.

### 2.3. Angle Random Walk (ARW)

Allan Variance is a classic method for separating and quantitatively identifying random noises of inertial devices. Various noise components such as angle random walk, bias instability and rate random walk can be distinguished according to the slope of curves plotted under double-logarithmic coordinates [17,19].

For the angular velocity sequence $\omega \left(t\right)$ of the gyroscope, continuous non-overlapping segmentation is first performed according to the cluster time τ:

(9) τ=mΔt

where *m* represents the number of data points in each segmented cluster, and Δt denotes the sampling period of the gyroscope. The value of *m* traverses the following range:

(10) $$m\to \left\{\begin{array}{cc} 1 & 2^{1} \end{array}\cdots \begin{array}{cc} 2^{k-1} & 2^{k} \end{array}\right\}$$

Until the following condition is satisfied:

(11) $$M=\frac{N}{m}\geq 2$$

Subsequently, the Allan Variance corresponding to the current τ is defined as:

(12) $$\sigma_{A}^{2}\left(\tau \right)=\frac{1}{2}\left\langle {\left({\bar{\omega }}_{k+1,m}-{\bar{\omega }}_{k,m}\right)}^{2}\right\rangle$$

where ${\bar{\omega }}_{k,m}$ is the average angular velocity within the *k*-th time window with duration τ; $\left\langle \cdot \right\rangle$ denotes the overall averaging operation.

In the double-logarithmic coordinate system constructed by ($\tau ,\;\sigma_{A}^{2}$), the slope of the typical Allan Variance curve $\sigma_{A}(\tau )$ corresponds to different types of noise: The segment with a slope of −1/2 corresponds to ARW; the segment with a slope of 0 corresponds to Bias Instability (BI); the segment with a slope of +1/2 corresponds to rate random walk (RRW).

Under low-speed vehicle dynamic conditions, excitations such as vehicle vibration and steering will significantly amplify the ARW component, extending the slope segment of −1/2 toward longer cluster times. Meanwhile, the flat region corresponding to bias instability exceeds the valid data length, which degrades the accuracy of the traditional joint identification method for ARW and BI. Therefore, only robust fitting is performed on the ARW component in this paper. On the Allan deviation curve, the longest continuous interval within the −1/2 slope range is first determined, and the calculation is conducted for each cluster time $\tau_{i}$ within this interval:

(13) $${ARW}_{i}=\sigma_{A}\left(\tau_{i}\right)\cdot \sqrt{\tau_{i}}$$

The arithmetic mean of all $A_{i}$ is taken as the estimated value of the ARW coefficient:

(14) $$\hat{ARW}=\left\langle {ARW}_{i}\right\rangle$$

where the unit of the estimated value is first converted to $rad/\sqrt{s}$, and then uniformly transformed into the commonly used engineering unit $deg/\sqrt{h}$.

### 2.4. Sub-Band Noise

To characterize the distribution characteristics of MEMS gyroscope error energy across different frequency scales, the Welch power spectral density (PSD) method is adopted for frequency-domain analysis in this paper [31,32]. By segmenting long time-series data, applying the Hanning window, and averaging the periodograms of each segment, the Welch algorithm can effectively reduce the spectral estimation variance of a single periodogram, which makes it suitable for frequency-domain analysis of long-term-measured data. Combined with the dynamic characteristics of low-speed vehicles and the sampling frequency, the full frequency range is divided into three characteristic frequency bands corresponding to different disturbance sources respectively:

(1) **Low-frequency band L (0.05–0.5 Hz)**: It corresponds to the slow variation in the gyroscope inherent bias and long-term low-frequency drift, matching the characteristics of low-speed vehicle maneuvering and long-term heading deviation.
(2) **Medium-frequency band M (0.5–5 Hz)**: It corresponds to vehicle body dynamic response, steering maneuvers, and suspension vibration, serving as the dominant excitation frequency band in the low-speed vehicle-mounted environment.
(3) **High-frequency band H (5–20 Hz)**: It corresponds to the inherent broadband noise of the sensor and local high-frequency vibration of the vehicle body.

For any frequency band $\left(f_{l},f_{h}\right)$, the root mean square (RMS) of noise within the band is calculated via integration of the power spectral density to characterize the total noise energy $P_{rms}$ of this frequency band:

(15) $$P_{rms}=\sqrt{\int_{f_{l}}^{f_{h}}S_{\omega }\left(f\right)df}$$

where $S_{\omega }(f)$ denotes the power spectral density function of the angular velocity error.

Meanwhile, the noise amplification ratio (NAR) is defined as the ratio of the RMS noise power under dynamic operating conditions to that under static operating conditions, so as to quantify the degradation degree of noise energy in each frequency band:

(16) $$P_{nar}=\frac{P_{rms}\left(dynamic\right)}{P_{rms}\left(static\right)}$$

## 3. Experiments and Results

### 3.1. Experimental Setup

**Figure 2:**
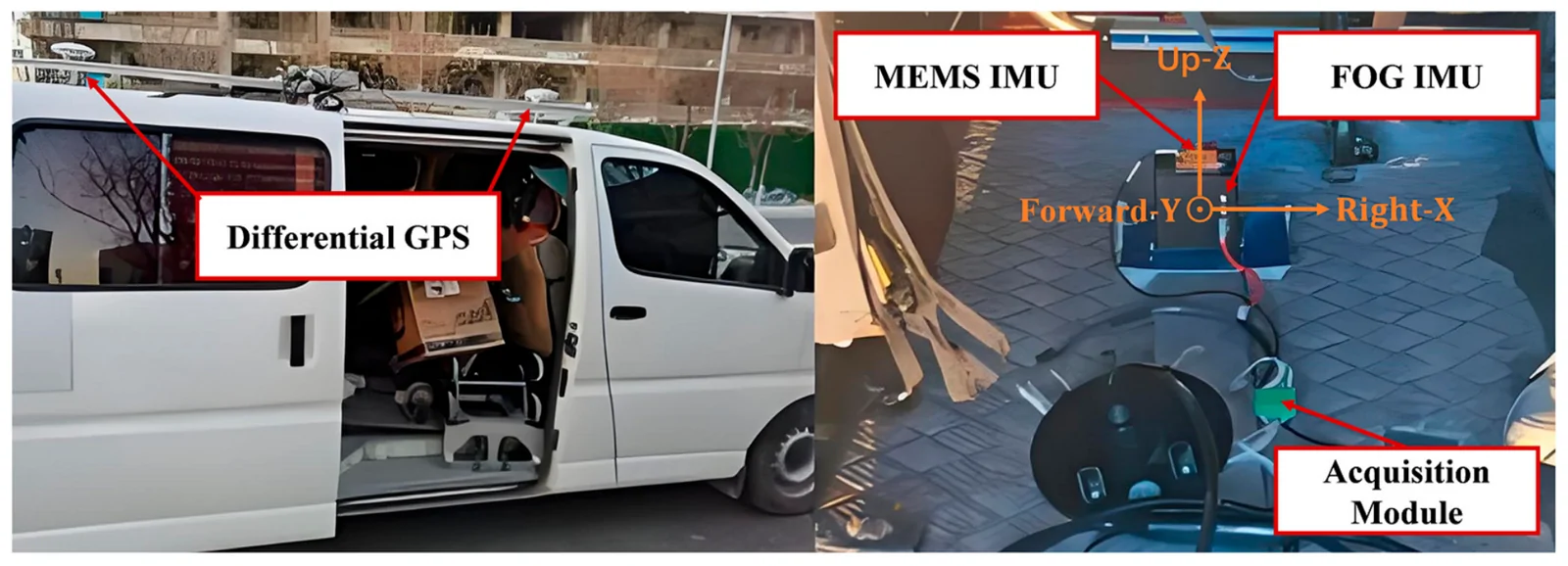
Experimental equipment.

To verify the method proposed in this paper, vehicle-borne data from the public Precise Strapdown Inertial Navigation System (PSINS) dataset are adopted for experimental analysis [33]. The vehicle-mounted test system in this experiment consists of four components: a MEMS inertial measurement unit, a navigation-grade fiber optic gyroscope, a differential GPS module, and an on-board data acquisition terminal. All sensors are rigidly mounted on the same installation plane of the test vehicle to ensure a consistent measurement reference for attitude and angular velocity, as illustrated in Figure 2. The three-dimensional coordinate axes (X-Y-Z) of the two IMUs are aligned with the vehicle’s right–forward–up orientation, respectively. The parameters and functions of each core device are described as follows:

(1) **MEMS IMU**: MTi-G-710, an industrial-grade commercial MEMS inertial device is taken as the test object in this experiment, with a triaxial angular velocity sampling frequency of 100 Hz. The typical MEMS gyro bias instability is 10°/h, and the angular velocity noise density is $0.01{}^{\circ}/s/\sqrt{Hz}$ [34].

(2) **FOG IMU**: A high-precision fiber optic gyroscope adopted as the reference benchmark for angular velocity measurement. It operates at a triaxial angular velocity sampling frequency of 100 Hz, featuring ultra-low static noise and favorable dynamic response performance. The typical bias instability of the gyroscope is less than 0.01°/h, and the angle random walk is no greater than 0.001${}^{\circ}/\sqrt{h}$.

(3) **Differential GPS**: It outputs data at a frequency of 5 Hz, providing vehicle position, driving speed, and heading angle information to classify motion intensity and resolve the vehicle motion state.
(4) **Data acquisition module**: It synchronously collects raw data from the three sensors, performs unified data storage and completes preliminary time sequence alignment based on GPS Pulse Per Second (PPS). Coordinate transformation of GPS data is accomplished based on lever-arm information obtained via offline measurement and calibration. The two IMUs undergo mechanical mounting and alignment. Since angular velocity and angular attitude serve as the major research objects in subsequent analysis, lever-arm errors exert negligible influences. An identity matrix is adopted to approximate the coordinate transformation throughout the analysis.

The test carrier employed in the experiment is a low-speed vehicle traveling entirely on paved roads. Its operating mode can simulate typical working conditions of low-speed mobile platforms such as autonomous delivery and park patrol inspection. The total duration of valid data collected in the complete vehicle test is approximately 3.3 h. Data are divided into the initial static phase and the long-term low-speed dynamic phase with the GPS velocity of 0.5 m/s set as the threshold:

(1) **Static phase:** The vehicle remains completely stationary for 730 s. This phase is used to collect static reference data of the MEMS gyroscope and FOG for initial bias calibration and static performance analysis.
(2) **Dynamic phase:** The vehicle travels continuously at a low-speed for a cumulative duration of approximately 3.0 h with a total driving mileage of around 10 km and an average speed of 12 km/h. The driving process covers multiple working conditions including straight travel, gentle turns, sharp turns, low-speed start and stop.

**Figure 3:**
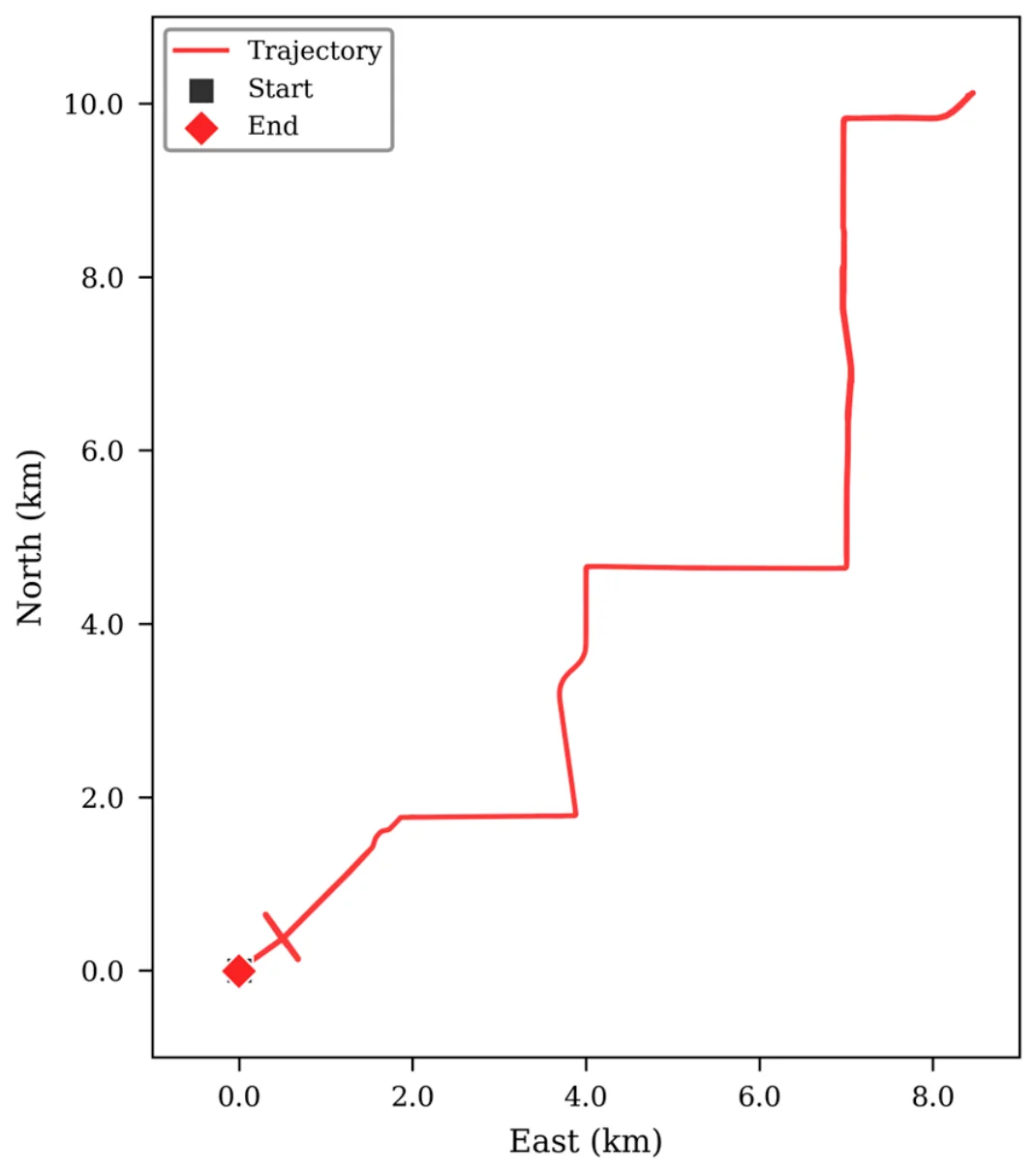
Motion trajectory of test data.

**Figure 4:**
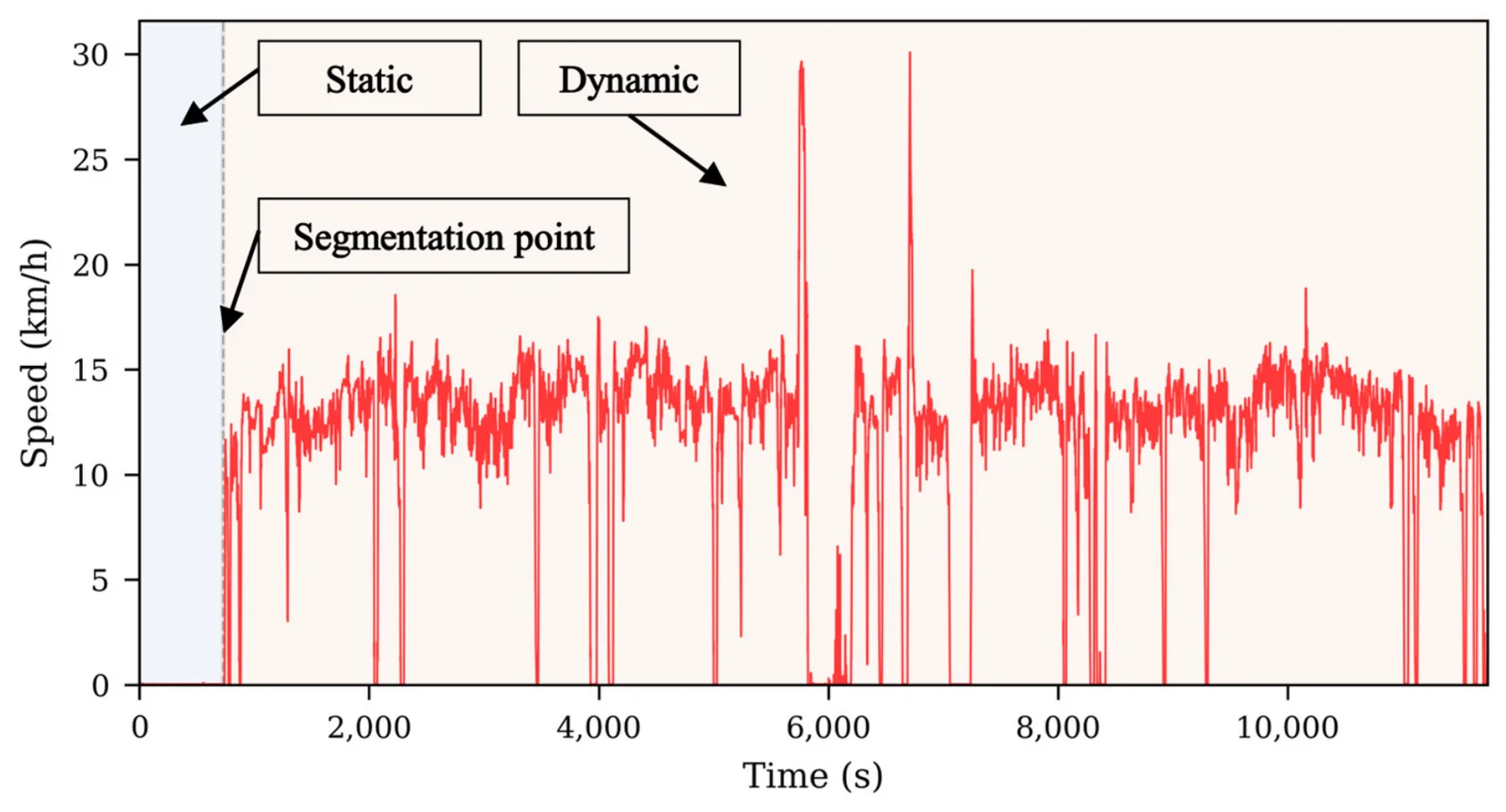
Time-varying velocity of test data.

**Figure 5:**
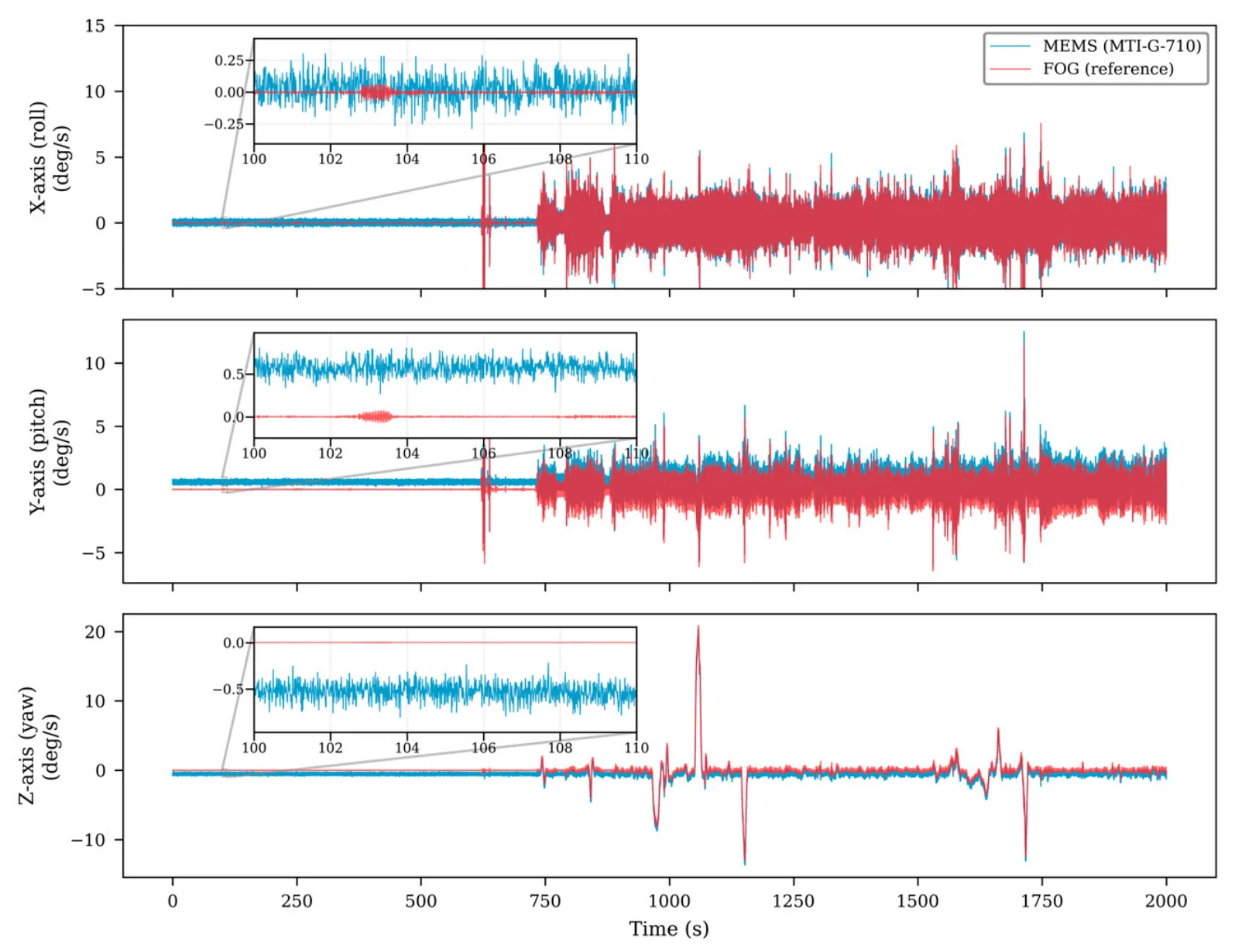
Gyroscope outputs of MEMS and FOG.

During the test, no extreme bumps or violent impacts occurred, which reproduces the typical daily operating environment of low-speed mobile platforms. The planar driving trajectory of the vehicle recorded by GPS is presented in Figure 3. The time-series velocity curve in Figure 4 can intuitively distinguish the static and dynamic phases as well as the vehicle motion states. The triaxial raw angular velocity data within the first 2000 s shown in Figure 5 compares the output characteristics of the MEMS gyroscope and the FOG. A locally enlarged segment of 10 s clearly reveals the output noise difference between the two types of sensors, which verifies the feasibility of adopting the FOG IMU as the reference benchmark.

### 3.2. Characteristics of Time-Domain Errors

**Figure 6:**
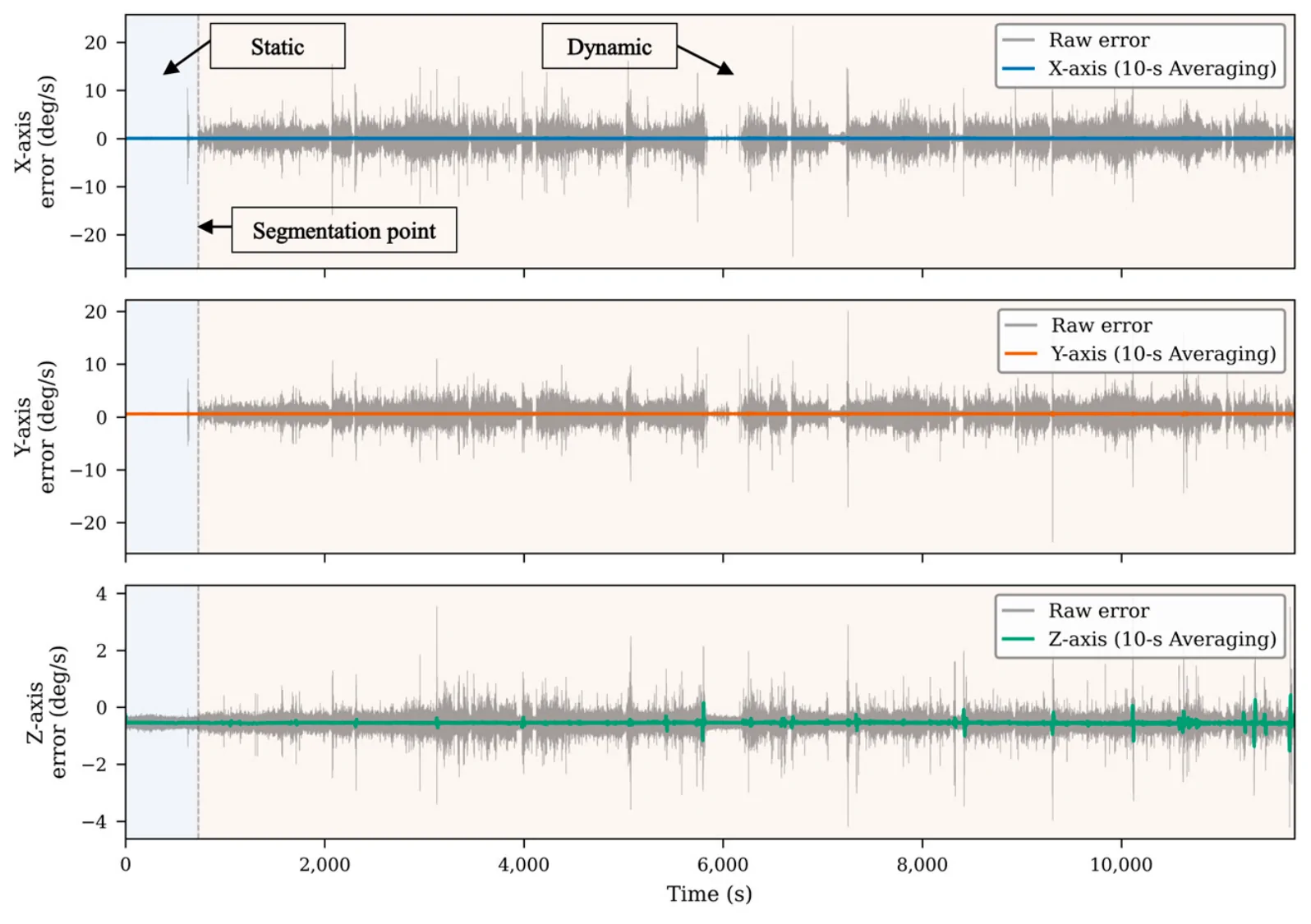
Error sequences of MEMS triaxial gyroscope.

Figure 6 illustrates the time-varying error curves of the MEMS triaxial gyroscope based on vehicle test data. During the stationary phase within the first 700 s, the triaxial angular velocity error sequences fluctuate slightly around zero with stable waveforms and low dispersion, where the errors only reflect the inherent static noise of the MEMS gyroscope. After 700 s in the motion phase, the dispersion degree of triaxial errors increases significantly, and error mutations and amplitude rise occur on different axes at different time intervals. This reveals that the noise amplification induced by low-speed vehicle motion is not uniformly distributed in the whole-time-domain; instead, it features temporal localization and axial differentiation, which is strongly correlated with transient operating conditions such as vehicle steering and vibration.

**Figure 7:**
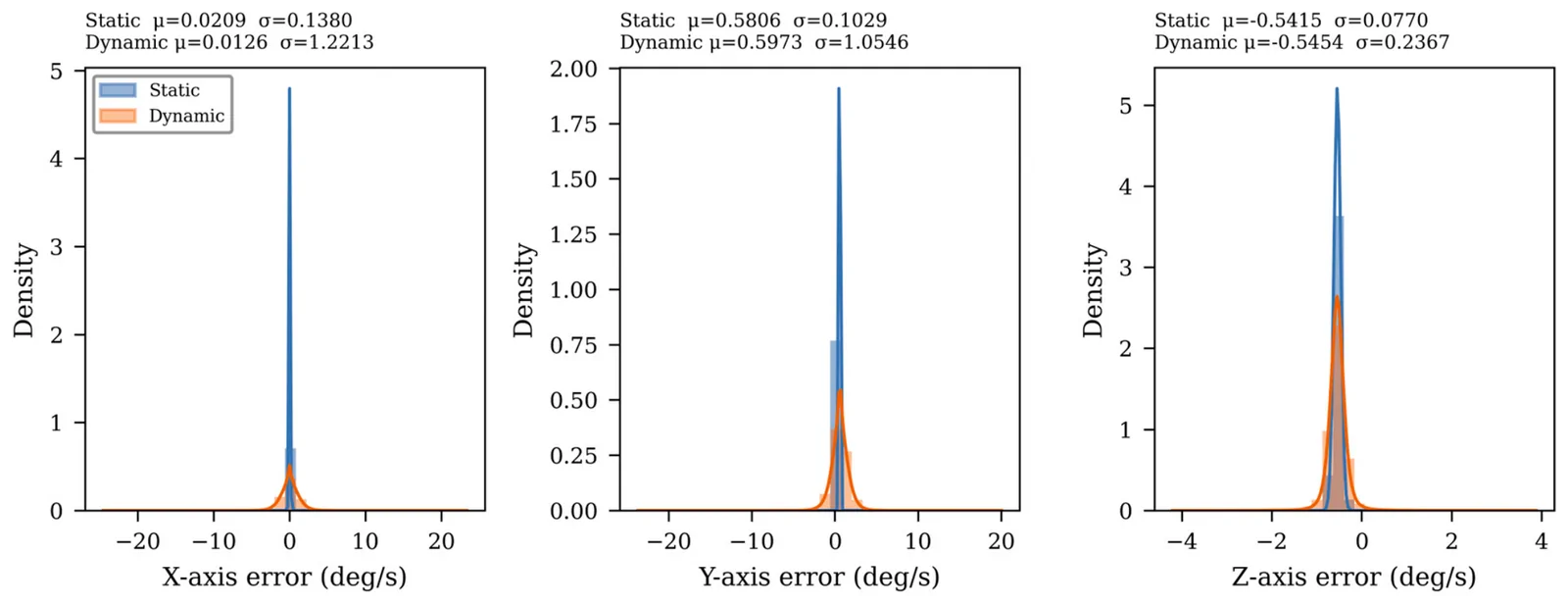
Error distribution of MEMS triaxial gyroscope.

Figure 7 shows the fitted Gaussian distribution probability density functions of the triaxial error sequences of the MEMS gyroscope under static and dynamic conditions. The mean values remain roughly consistent with minor fluctuations between the two working conditions, while the horizontal distribution range under dynamic operation is significantly wider than that in the static state. The standard deviations of the three axes under dynamic conditions are 1.22°/s, 1.05°/s and 0.24°/s respectively, which increase by 8.9 times, 10.2 times and 3.1 times compared with those under static conditions.

### 3.3. Long-Term Cumulative Drift Analysis

The triaxial cumulative attitude drift is obtained by integrating the high-frequency angular velocity errors after removing the static mean value, and linear fitting is carried out for the static segment and dynamic segment respectively. The fitting parameters and characteristic analysis are presented as follows: both the MEMS and FOG IMUs are processed by eliminating the initial static mean value to remove the bias repeatability error caused by repeated power-on operations.

**Figure 8:**
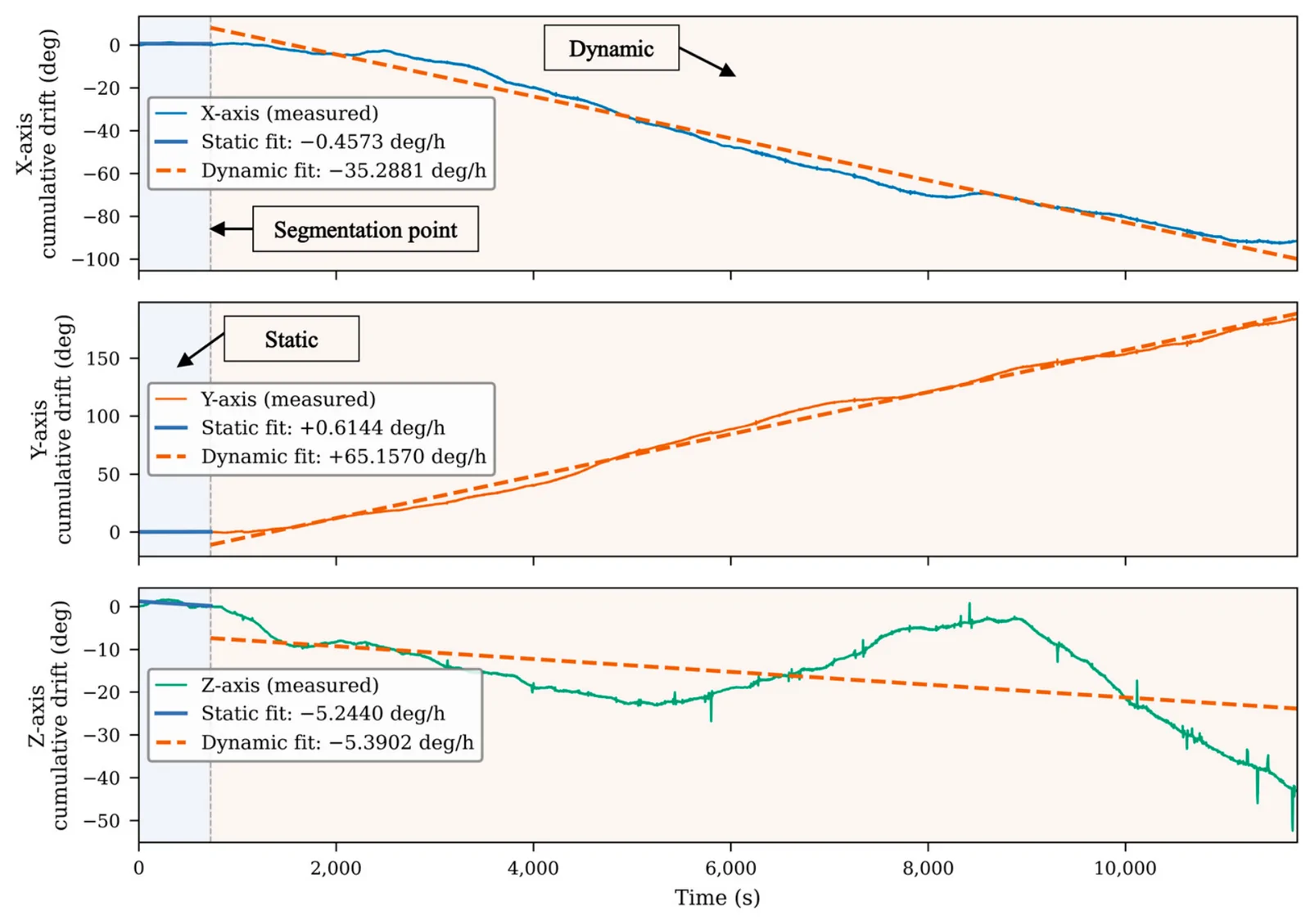
Long-term cumulative drift of MEMS gyroscope errors.

In Figure 8, under static conditions, the drift slopes of the *X*-, *Y*-, and *Z*-axes are −0.46°/h, 0.61°/h and −5.24°/h in sequence. Overall, the drift slopes in the static phase are extremely small, and the cumulative attitude error increases slowly. The MEMS IMU exhibits excellent relative error stability within a short period, which is consistent with the ideal characteristics obtained from laboratory static calibration.

Under dynamic operating conditions, the drift slopes of the *X*-, *Y*-, and *Z*-axes change to −35.29°/h, 65.16°/h and −5.39°/h respectively. The dynamic drift slopes of the *X* and *Y* axes increase by more than two orders of magnitude compared with their static counterparts, with linear fitting coefficients of determination of 0.9821 and 0.9935, indicating strong linear correlation. This demonstrates that the two horizontal axes suffer from a stable long-term linear drift trend during vehicle operation. In contrast, the dynamic drift slope of the *Z*-axis is basically consistent with its static value; yet, the corresponding coefficient of determination is only 0.2386, which reflects a weak linear characteristic. It can be concluded that the cumulative drift of the *Z*-axis is not dominated by long-term slow drift, but is mainly governed by transient disturbances such as local vehicle maneuvers and nonlinear vibrations.

### 3.4. Bias Stability Analysis

The bias stability indexes of the MEMS gyroscope are calculated by the 10 s averaging method under static and dynamic conditions respectively, and the detailed results are listed in Table 1. The bias stability of all three axes increases remarkably in the dynamic scenario. Specifically, the values of the *X*-axis and *Y*-axis rise by 2.1 times and 2.7 times correspondingly. The *Z*-axis witnesses the most significant increment, with its dynamic bias stability reaching 230.08°/h, which is 7.7 times higher than the static value.

**Figure 9:**
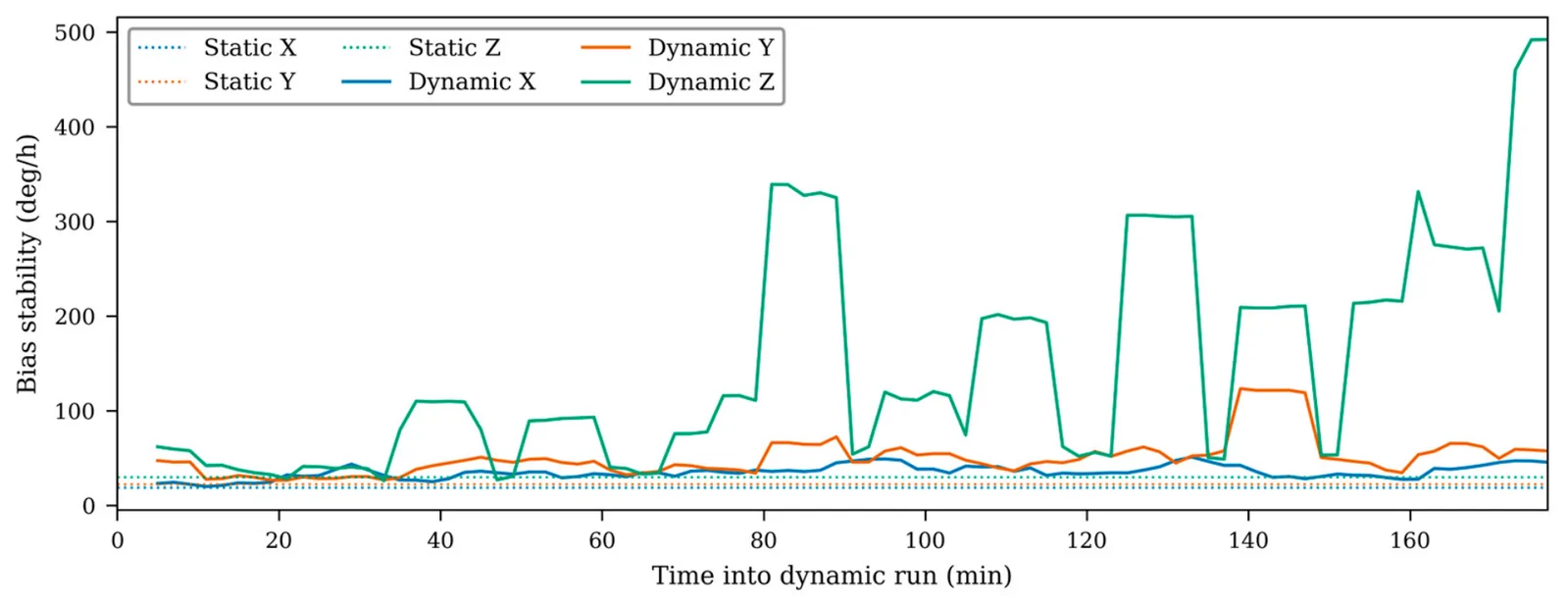
Time-varying bias stability under dynamic motion conditions.

The time-varying bias stability under dynamic conditions is continuously calculated using a 10 min sliding window combined with the 10 s averaging method, and the corresponding results are illustrated in Figure 9. The dashed line in the figure denotes the static bias stability, while all solid curves under dynamic conditions are markedly higher than the static reference value. This reveals that the bias error of the MEMS gyroscope during motion differs significantly from that in the static state, and direct reuse of static calibration parameters will lead to larger cumulative errors. Meanwhile, it can be observed that the triaxial bias stability fluctuates continuously with the vehicle driving time rather than remaining constant. The fluctuation range of the *Z*-axis is far larger than those of the *X*- and *Y*-axes, and the mutation time intervals are correlated with vehicle operating conditions, demonstrating that varying motion intensity serves as one of the major excitation sources for the aggravated *Z*-axis bias error.

**Figure 10:**
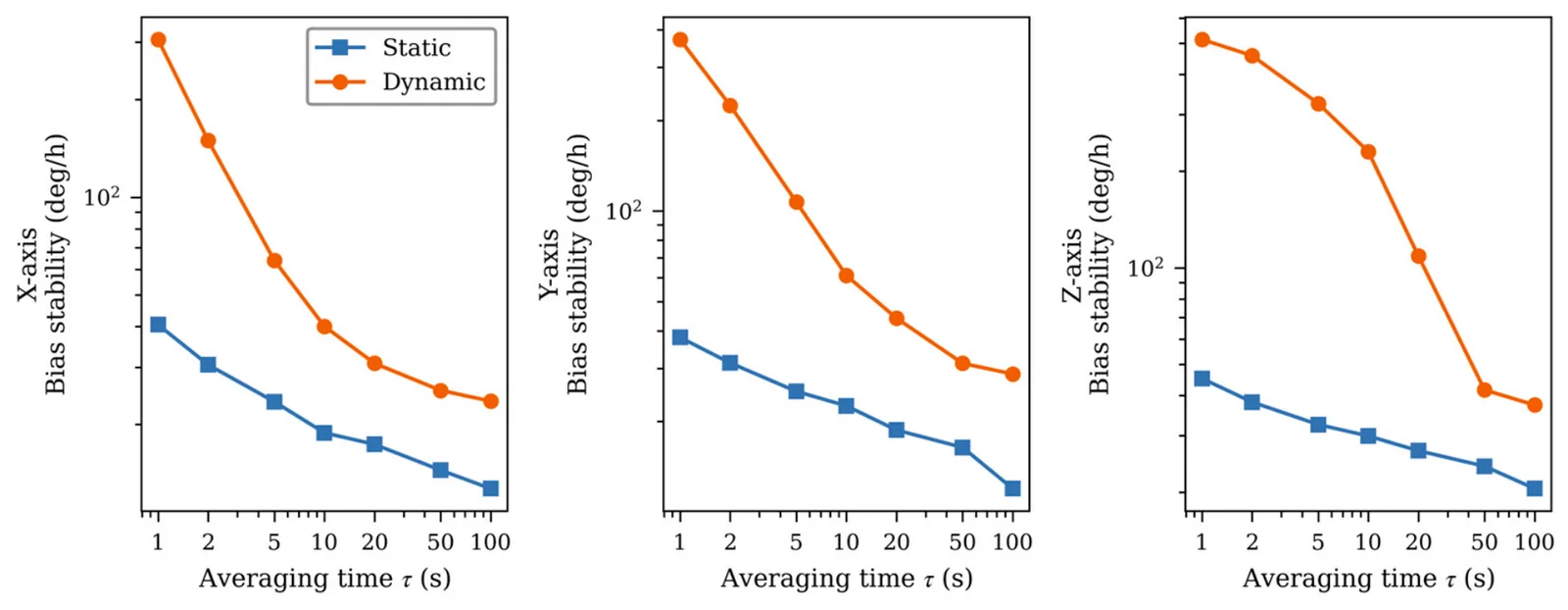
Bias stability under different smoothing durations.

To further investigate the bias stability indicators under different smoothing durations, the bias stability values are sequentially calculated at averaging windows of 1 s, 2 s, 5 s, 10 s, 20 s, 50 s and 100 s, as presented in Figure 10. As the smoothing time increases, short-term high-frequency noise is suppressed to varying degrees, and the bias stability gradually improves under both static and dynamic conditions. Nevertheless, the dynamic bias stability remains consistently inferior to the static counterpart across all time scales. This indicates that continuous vehicle motion exerts adverse impacts on both short-term high-frequency noise and long-term low-frequency bias, degrading the gyroscope error performance over the entire time range.

### 3.5. ARW Analysis

**Figure 11:**
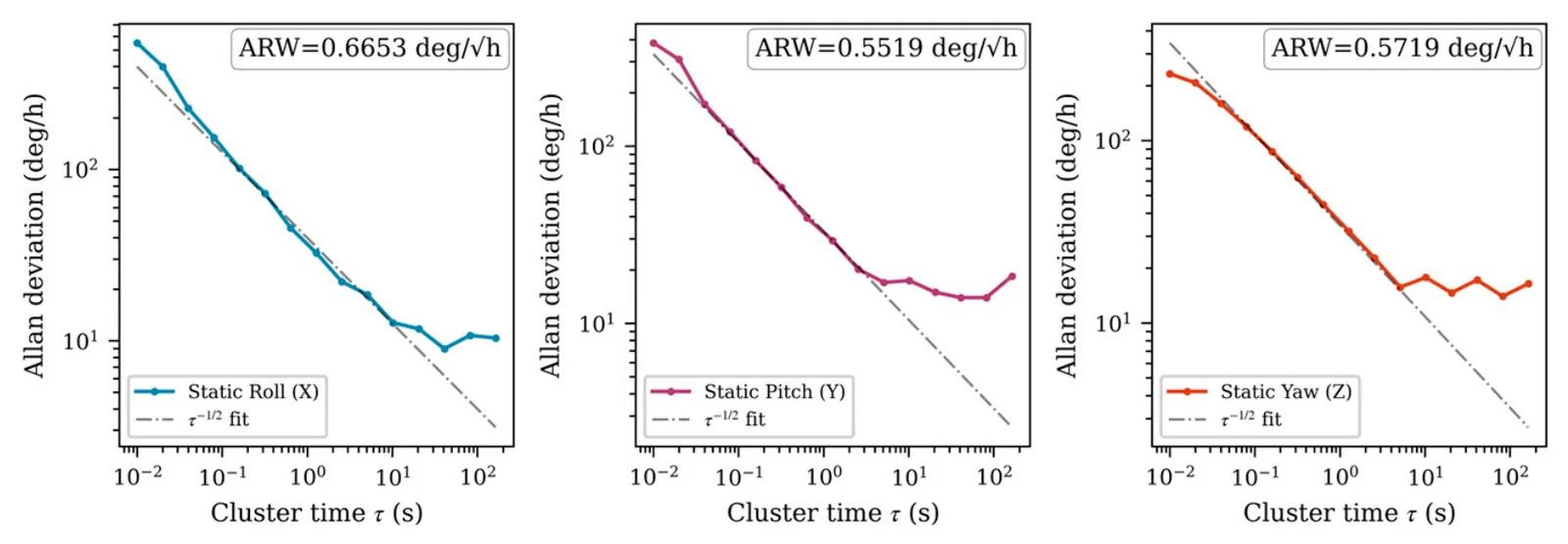
ARW of MEMS gyroscope under static condition.

**Figure 12:**
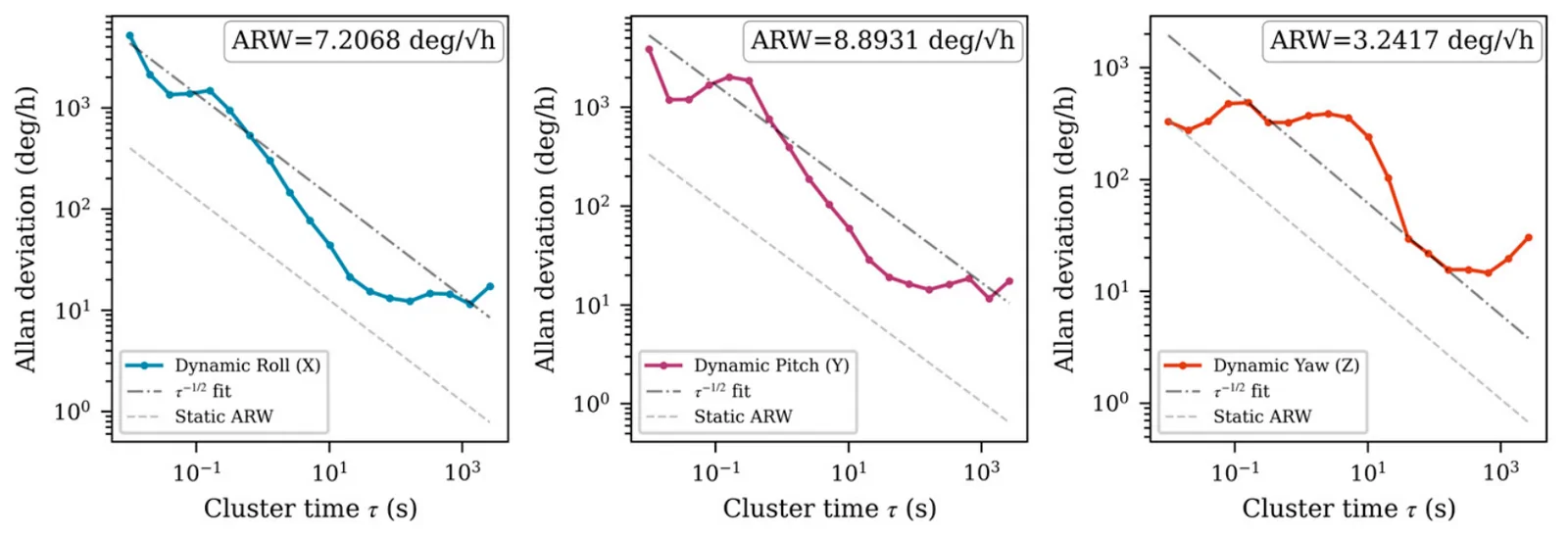
ARW of MEMS gyroscope under dynamic condition.

Allan Variance is adopted to analyze the MEMS gyro error sequences under static and dynamic conditions, with the corresponding results shown in Figure 11 and Figure 12, respectively. The Allan Variance curves under static conditions remain relatively stable, whereas those obtained under dynamic conditions lie entirely above their static counterparts, indicating a significant degradation of gyroscope stability under motion. Allan Variance is calculated at equal intervals of $2^{k}$ on the log–log coordinate plane, with the upper limit of cluster time set to one quarter of the total data duration. ARW is extracted by identifying the longest continuous segment with a local slope close to −1/2. The average value is calculated from all points within this segment.

By fitting the segment with a slope of −1/2 from the static curves, the static ARW coefficients of the three axes are obtained as follows: 0.67°/√h for the *X*-axis, 0.55°/√h for the *Y*-axis and 0.57°/√h for the *Z*-axis. The three axes exhibit close ARW values with an overall low level of random walk noise under static conditions, which is consistent with the static parameters provided in the device datasheet [34].

The triaxial dynamic ARW coefficients are further derived by fitting the MEMS gyro error sequences collected from the entire 3 h continuous dynamic test, and the corresponding degradation ratios are summarized in Table 2. The ARW coefficients of all three axes degrade by an order of magnitude under dynamic operating conditions, among which the *Y*-axis suffers the most severe amplification with a degradation factor of 16.2 times.

As can be observed from the ARW results of the *X*- and *Y*-axes in Figure 12, the dynamic Allan Variance curves maintain a slope of −1/2 over a wide range of cluster times, while the flat region corresponding to BI almost disappears. This phenomenon originates from the sharp increase in the amplitude of angle random walk under dynamic conditions, whose dominant interval extends toward longer cluster times. Within the valid 3 h dataset, the low-frequency characteristic of bias instability can hardly be identified.

The Allan Variance curve of the dynamic *Z*-axis shows a transition trend from a slope of −1/2 to zero with an emerging BI flat region, which also verifies that the ARW degradation degree of the *Z*-axis is relatively mild. It can be concluded that the static calibrated bias instability parameters can barely be accurately identified in the dynamic vehicle-mounted environment. Directly adopting static BI parameters for navigation filter design will overestimate sensor performance and further cause inaccuracy of the filtering algorithm.

### 3.6. PSD and Sub-Band Noise Analysis

**Figure 13:**
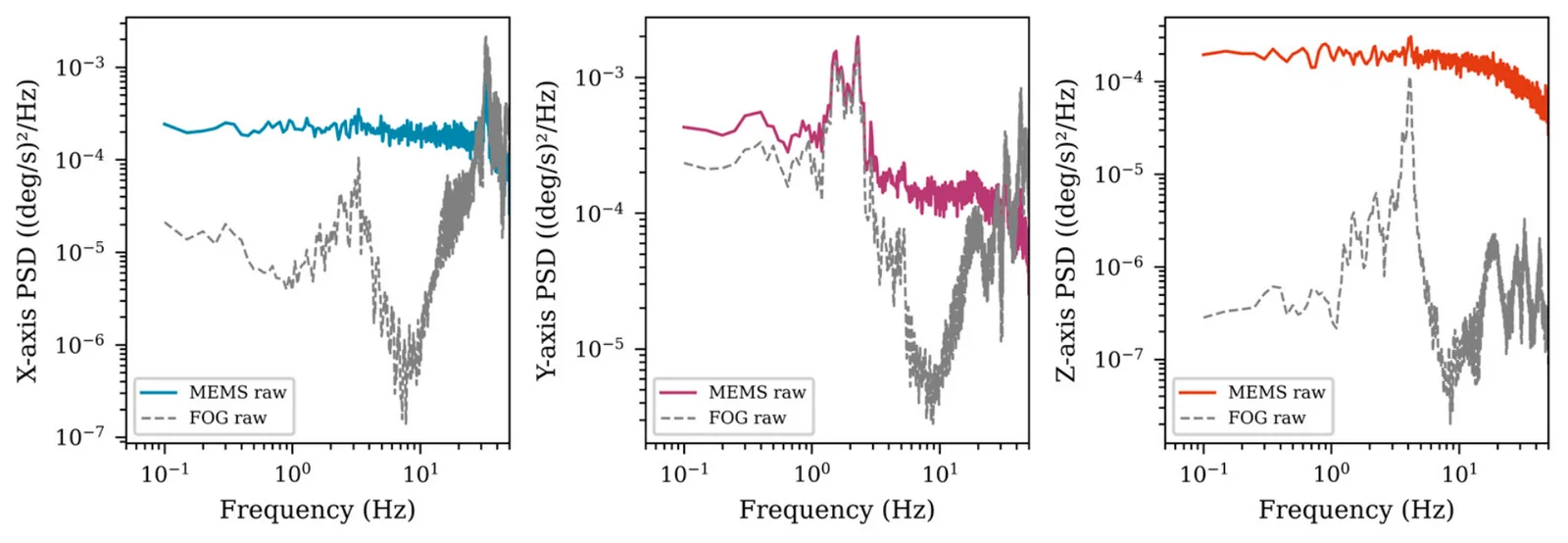
PSD comparison between MEMS gyroscope and FOG.

Figure 13 presents the comparison of the PSD of raw outputs from MEMS gyroscope and FOG obtained via the Welch method. PSD estimation is implemented via the Welch algorithm with a Hanning window. The window length is set to 20 s with 50% overlap, and the frequency range covers 0.05–50 Hz. The full spectrum is divided into three characteristic frequency bands: low-frequency band L (0.05–0.5 Hz), mid-frequency band M (0.5–5 Hz), and high-frequency band H (5–20 Hz). This classification is mainly implemented according to classical literature on vibration and spectrum analysis within the field of vehicle dynamics [35,36]. The integrated RMS noise of each band is adopted as the quantitative indicator. For sliding-window RMS analysis, a 40 s window and 10 s step size are utilized. Welch estimation and subsequent integration are conducted window by window. The results show the PSD of the MEMS gyroscope is higher across the entire frequency band, with particularly prominent discrepancies in the low- and medium-frequency ranges, which reflects the inherent characteristic of relatively large low-frequency noise for MEMS devices. In the high-frequency band, the noise density of the two types of gyroscopes is close to each other, revealing the consistent high-frequency sensing characteristics of gyroscopic sensors. In static conditions, sensor noise is predominantly concentrated in the low-frequency range. The PSD curve of the FOG is evidently much lower than that of the MEMS IMU, which further verifies the rationality of adopting the FOG as the reference sensor in this paper.

**Figure 14:**
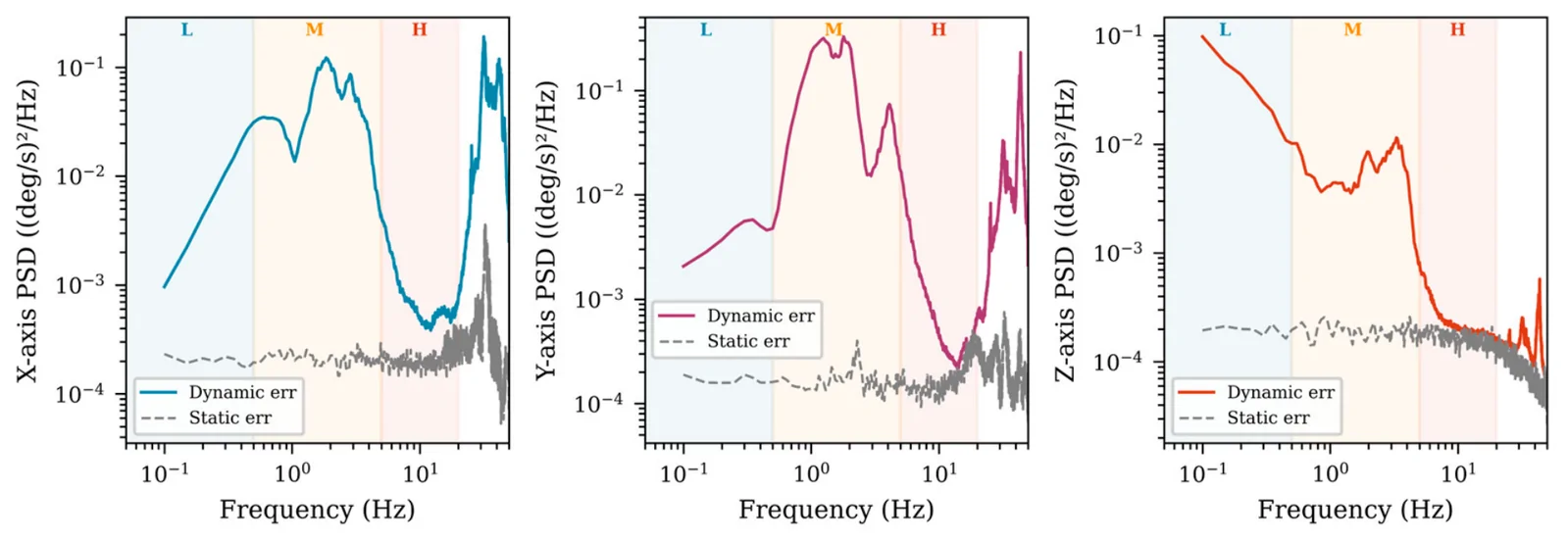
PSD of MEMS gyroscope under dynamic and static conditions.

Figure 14 shows the power spectra of the MEMS gyroscope error sequences under static and dynamic operating conditions. The spectral density under dynamic conditions is higher across the entire frequency band compared with the static case, and the most significant amplification occurs in the intermediate frequency band (0.5–5 Hz). In practical applications, noise analysis and modeling for this frequency range should be prioritized.

The statistical results of total noise energy obtained by integrating the triaxial error sequences of the MEMS gyroscope in different frequency bands and the amplification ratios between dynamic and static conditions are summarized in Table 3. According to the results:

In the mid-frequency band (0.5–5 Hz), all three axes exhibit the maximum amplification factor of noise energy, with the *Y*-axis reaching 24.7 times. This frequency band overlaps with the characteristic frequency range corresponding to dynamic responses of the vehicle suspension system and mechanical vibrations of the steering mechanism. It is inferred that this band acts as one of the potential dominant bands for low-frequency disturbances on the experimental platform, and may serve as the primary source triggering noise performance deterioration of MEMS gyroscopes under vehicle-mounted operating conditions. Nevertheless, accurate vibration source identification requires synchronous vibration measurement by mounting accelerometers on key vehicle positions in follow-up research. Such tests help eliminate coupled interference induced by engine harmonics, random road excitations and other disturbances, so as to further confirm the dominant mechanism of this frequency band.

In the low-frequency band (0.05–0.5 Hz), the amplification ratio ranges from 5.2 to 12.8 times, and the *Z*-axis exhibits a low-frequency amplification of 12.8 times, which is directly associated with long-duration vehicle steering and low-frequency heading drift.

In the high-frequency band (5–20 Hz), the noise energy amplification ratio falls between 1.2 and 2.4 times with slight performance degradation, indicating that low-speed movement exerts little influence on the inherent high-frequency noise of the gyroscope.

**Figure 15:**
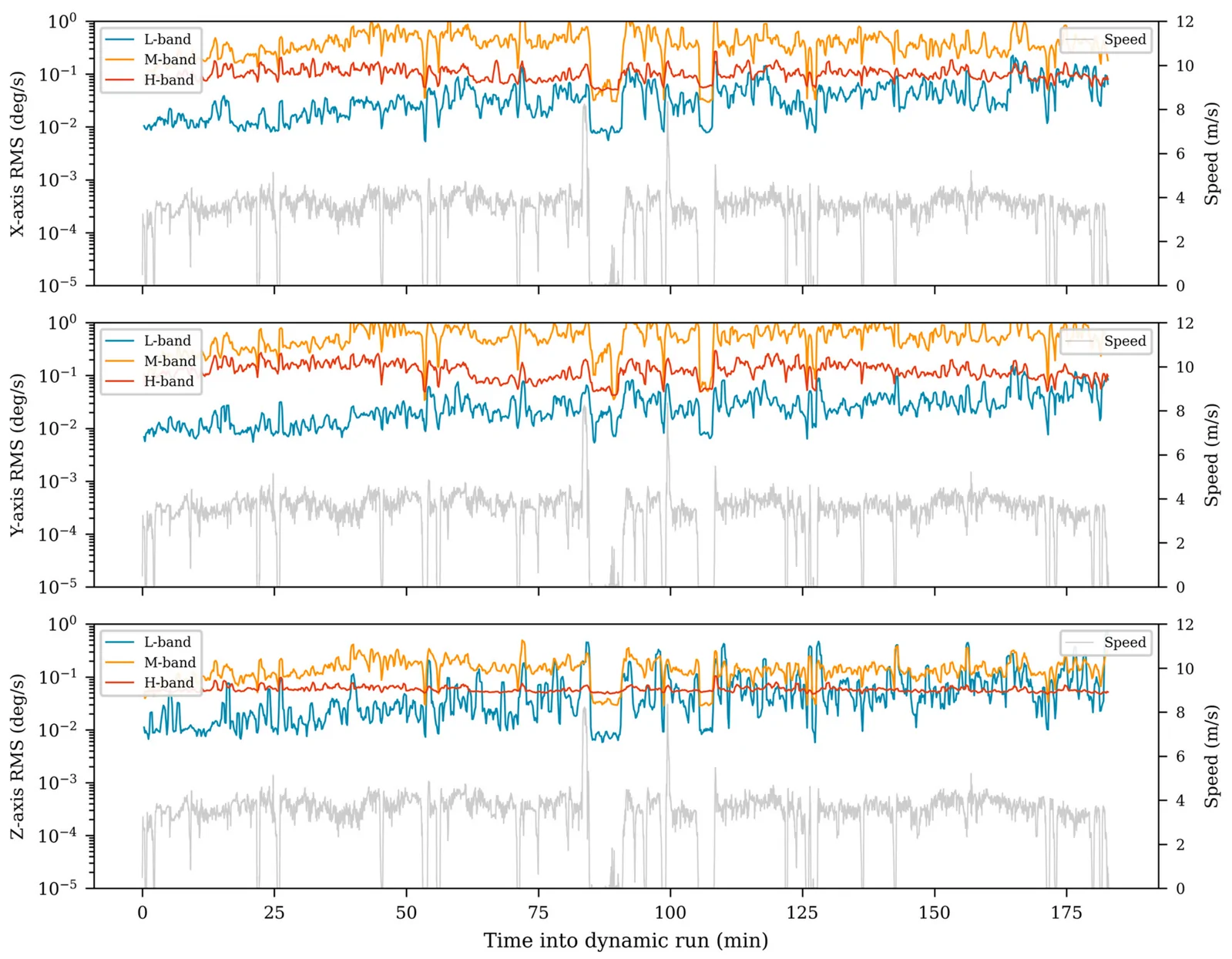
Time-varying sub-band noise energy curves under dynamic conditions.

To conduct a detailed analysis of time-series noise characteristics in different frequency bands, sliding windows are utilized to generate the time series of root mean square power $P_{rms}$ for each frequency band, which are then visualized together with the GPS speed curve for comparative analysis, as shown in Figure 15. The results reveal that the noise energy of each frequency band for all three axes fluctuates in real time along with vehicle speed and motion states. The temporal variation in intermediate-frequency $P_{rms}$ is highly synchronized with vehicle speed and steering maneuvers, further verifying that vehicle dynamic vibration serves as the direct inducement of intermediate-frequency noise. The intermediate-frequency noise energy of the *Y*-axis is an order of magnitude higher than that of other frequency bands during certain periods, demonstrating that the test vehicle possesses the strongest intermediate-frequency vibration energy in the pitch direction.

## 4. Discussion

Combining the experimental results from the time-domain, Allan Variance domain and frequency-domain, this paper comprehensively analyzes the evolution mechanism of dynamic noise for MEMS gyroscopes under low-speed vehicle motion, and investigates the coupling relationship between external excitations and sensor errors.

The influence of vehicle motion on MEMS gyro errors lies in the non-uniform multidimensional reconstruction across all time scales, instead of simple amplification of noise amplitude. MEMS gyroscopes feature stable noise structures and fixed parameters under static conditions. Once operating in dynamic scenarios, external disturbances including road excitations, suspension vibrations and steering maneuvers act on the sensor simultaneously across three frequency bands: low-frequency drift, medium-frequency vibration, and high-frequency noise. These disturbances not only raise the overall noise intensity, but also thoroughly change the temporal and frequency distribution characteristics of errors. Traditional single-parameter models based on static calibration cannot adapt to dynamic operating conditions at all.

There exist remarkable discrepancies in the dominant error mechanisms among different axes. The horizontal *X*- and *Y*- axes are most severely affected by vehicle medium-frequency vibration, accompanied by the highest degradation of angle random walk and strongly linear long-term drift. As the heading axis, the *Z*-axis is sensitive to vehicle steering, scale factor errors and residual lever-arm effects, suffering the most serious degradation of bias instability with cumulative drift dominated by nonlinear disturbances. The differentiated error characteristics of the three axes are jointly determined by the sensor mounting orientation and the directional coupling of vehicle dynamic excitations.

Variations in Allan Variance characteristics reveal the succession of dominant and subordinate noise components. Under dynamic conditions, the drastically amplified angle random walk becomes the dominant noise, covering the originally identifiable bias instability. This explains why static bias instability parameters are poorly matched with dynamic scenarios. If navigation filters still adopt static bias instability parameters, low-frequency dynamic errors will be underestimated, further leading to deviations in attitude calculation.

Frequency-domain characteristic analysis further clarifies the technical route for error suppression. Under the experimental conditions of this work, the mid-frequency band exhibits the most prominent increase in noise energy. It is inferred that this phenomenon is correlated with mechanical vibrations during vehicle travel, including dynamic responses of the suspension system and excitations from steering actuators. This frequency band constitutes one of the dominant contributing bands for dynamic errors of MEMS gyroscopes in vehicle-mounted scenarios. The noise within this band can be suppressed via engineering approaches such as structural vibration reduction and mid-frequency band-stop filtering. High-frequency noise is barely affected by vehicle motion, thus no targeted processing is needed.

A coupling rule exists between motion intensity and sensor errors. The overall noise level remains low when the vehicle travels straight; gentle turns only slightly amplify triaxial noise, while sharp turns will trigger abrupt errors on the *Z*-axis. In the design of control and navigation algorithms for low-speed vehicles, turning intensity can be introduced as a state variable to implement adaptive compensation for *Z*-axis heading errors.

Based on the dynamic noise analysis method proposed in this paper, a motion-state-driven band-divided adaptive compensation strategy can be developed for practical engineering applications in follow-up research. This strategy can adjust the error compensation weight of each frequency band in real time according to the carrier motion state, converting band-split noise into state-dependent compensation parameters. For instance, calibration for low-frequency drift of the *Z*-axis is strengthened during steering maneuvers, while mid-frequency vibration suppression is prioritized under uniform driving conditions. This framework delivers feasible engineering implementation ideas for dynamic error compensation of MEMS gyroscopes applied to low-speed mobile platforms.

Nevertheless, the current analysis relies on experimental data collected from only a single test vehicle, so the present study has certain limitations that require further refinement in follow-up research. The quantitative conclusions obtained in this paper mainly characterize observations under the specific experimental configuration adopted herein; their statistical significance and generalizability remain to be further validated. Accordingly, caution should be exercised when extrapolating the present quantitative results to other types of MEMS gyroscopes, diverse vehicle platforms, or more complex driving scenarios. Future work will conduct repeated experiments across multiple vehicles and multiple sensor devices alongside long-timescale data acquisition. Statistical uncertainty analysis approaches including confidence interval evaluation and hypothesis testing will be adopted to further quantify the credible range of conclusions, thereby improving the generalization performance and engineering guiding value of the research outcomes.

## 5. Conclusions

This paper establishes a joint analytical framework integrating time-domain analysis, Allan Variance analysis, and frequency-domain analysis. With a navigation-grade FOG adopted as the motion reference benchmark, this study preliminarily realizes effective decoupling between the true carrier motion under low-speed vehicle-borne conditions and the inherent errors of MEMS gyroscopes. According to measured experimental data acquired in this work and within the operating conditions covered by the experiments, noise amplification of the MEMS gyroscope fails to exhibit simple proportional growth relative to static noise; instead, it presents complicated frequency selectivity and axial differentiation features. This paper conducts preliminary analysis and discussion on the above phenomenon from multiple dimensions.

Combined static and dynamic vehicle tests reveal the following major phenomena under the experimental conditions: (1) Obvious axial anisotropy exists in the time-domain. The degradation factor of 10 s bias stability ranges approximately from 2.1 to 7.7. The heading axis shows non-stationary degradation strongly correlated with steering intensity, while the horizontal axes mainly follow a steady long-term linear drift trend. (2) Dynamic excitations cause the angle random walk (ARW) coefficient to increase by roughly 5.7–16.2 times. Meanwhile, amplified noise extends to longer correlation times, nearly masking the static bias instability features on the Allan Variance curves obtained in this experiment. This finding helps explain why parameters calibrated under static conditions cannot be directly reused in dynamic applications. (3) Noise deterioration possesses prominent frequency-band selectivity. Noise energy in the mid-frequency band correlated with vehicle vibration characteristics in this experiment rises by a factor of 5.5–24.7, which is inferred as the dominant frequency band triggering dynamic performance degradation. By contrast, the high-frequency band is comparatively less affected by carrier motion.

Based on the above observations, this paper suggests that engineering practice should gradually shift away from evaluation schemes merely dependent on static calibration toward performance evaluation oriented to practical dynamic operating conditions. Device performance assessment, parameter tuning, and error compensation implemented under real dynamic scenarios can better exploit the application potential of MEMS gyroscopes in low-speed intelligent mobile devices.

The analytical framework proposed in this paper provides a feasible technical reference for investigating dynamic characteristics of inertial sensors applied to low-speed intelligent mobile platforms. Building on existing outcomes, follow-up research will further explore stochastic error modeling and dynamic calibration methods oriented to motion perception. Multiple-device repeated tests and statistical uncertainty analysis will be adopted to verify and revise the preliminary conclusions drawn in the current study.

## Figures and Tables

**Table 1:** Bias stability results via 10 s averaging method under static and dynamic conditions.

10 s Averaging Method	*X*-Axis (°/h)	*Y*-Axis (°/h)	*Z*-Axis (°/h)
Static	18.85	22.54	29.93
Dynamic	40.07	61.10	230.08
Increase multiple	2.1	2.7	7.7

**Table 2:** ARW degradation ratio under static and dynamic conditions.

Axis	Static (°/√h)	Dynamic (°/√h)	Degradation Ratio
X	0.67	7.21	10.8
Y	0.55	8.89	16.2
Z	0.57	3.24	5.7

**Table 3:** Total noise energy of triaxial MEMS gyroscope in different frequency bands.

Axis	Frequency Band	Static $P_{rms}$ (deg/s)	Dynamic $P_{rms}$ (deg/s)	$P_{nar}$
X	Low	0.32	2.58	8.0
	Medium	1.10	16.36	14.8
	High	2.05	3.86	1.9
Y	Low	0.30	1.52	5.2
	Medium	0.98	24.24	24.7
	High	1.98	4.83	2.4
Z	Low	0.32	4.06	12.8
	Medium	1.05	5.73	5.5
	High	1.77	2.10	1.2

## Data Availability

The original data presented in the study are openly available in https://psins.org.cn/dhsj (accessed on 12 May 2026).

## Abbreviations

The following abbreviations are used in this manuscript:

MEMS	Micro-Electro-Mechanical System
ARW	Angle Random Walk
BI	Bias Instability
RRW	Rate Random Walk
PSD	Power Spectral Density
